# Murine transcription factor Math6 is a regulator of placenta development

**DOI:** 10.1038/s41598-018-33387-x

**Published:** 2018-10-09

**Authors:** Marion Böing, Beate Brand-Saberi, Markus Napirei

**Affiliations:** Ruhr University Bochum, Institute of Anatomy, Department of Anatomy and Molecular Embryology, Bochum, Germany

## Abstract

The murine basic helix-loop-helix transcription (bHLH) factor mouse atonal homolog 6 (Math6) is expressed in numerous organs and supposed to be involved in several developmental processes. However, so far neither all aspects nor the molecular mechanisms of Math6 function have been explored exhaustively. To analyze the *in vivo* function of Math6 in detail, we generated a constitutive knockout (KO) mouse (*Math6*^−/−^) and performed an initial histological and molecular biological investigation of its main phenotype. Pregnant *Math6*^−/−^ females suffer from a disturbed early placental development leading to the death of the majority of embryos independent of the embryonic *Math6* genotype. A few placentas and fetuses survive the severe uterine hemorrhagic events at late mid-gestation (E13.5) and subsequently develop regularly. However, these fetuses could not be born due to obstructions within the gravid uterus, which hinder the birth process. Characterization of the endogenous spatiotemporal *Math6* expression during placenta development reveals that Math6 is essential for an ordered decidualization and an important regulator of the maternal-fetal endocrine crosstalk regulating endometrial trophoblast invasion and differentiation. The strongly disturbed vascularization observed in the maternal placenta appears as an additional consequence of the altered endocrine status and as the main cause for the general hemorrhagic crisis.

## Introduction

At the beginning of mammalian life, the implantation of a fertilized blastocyst and the ordered development of a placenta are necessary for the establishment of a successful and productive pregnancy. The placenta connects the developing embryo to the maternal uterus and provides nutrients and oxygen exchange via the maternal blood supply. Any impairment of the strictly regulated placental development could lead to pregnancy complications or miscarriage^1^.

The rodent uterus consists of two horns each of which is supplied from an *Arteria ovarica* and an *Arteria uterina* situated in the mesometrium. The innermost layer of the uterus wall is the endometrium, which consists of an epithelial cell layer and the stroma containing the uterine glands. The adjacent layer is the myometrium composed of smooth muscle tissue and covered by the perimetrium^2^.

After the fertilization, the trophoblast of the blastocyst attaches to the endometrial epithelium and implantation proceeds at the anti-mesometrial side. The mural trophectoderm proliferates and gives rise to all kinds of trophoblast cells, which build up the fetal part of the placenta. In mice, the attachment of the blastocyst to the epithelium initializes the decidualization. During decidualization, the endometrial stroma cells proliferate and remodel their morphology by a MET (mesodermal-epithelial transition)-like process to epithelial-like cells called decidual cells^3^. The decidua regulates the invasion of trophoblasts and promotes immunological tolerance towards fetal cells by secretion of cytokines^4^. At day E10.5 the phase of mid-gestation starts and all layers of the definitive chorioallantoic placenta are established consisting of the maternal part, called decidua, and the fetal part composed of the junctional zone with trophoblast giant cells, spongiotrophoblasts and glycogen trophoblasts, the labyrinth and the chorionic trophoblast at the base of the forming labyrinth. The labyrinth generates the hemotrichorial placental barrier for nutrient exchange and is formed by syncytiotrophoblasts I and II as well as by fetal endothelial cells^5,6^. At day E13.5 mid-gestation ends with the termination of embryonic development and the fetus matures up to the time of birth around day E19.5^7^.

For a successful pregnancy in humans as well as in mice, remodeling of the maternal blood vessels is very important to supply the increasing need of nutrition for the growing embryo and fetus. Indeed, a disarranged adjustment of the blood vessel system leads to pregnancy disorders like pre-eclampsia or intrauterine growth restriction^8^. During the process of remodeling, the uterine blood vessels and the endometrial spiral arteries are enlarged. Two characteristic features of spiral artery remodeling are the degradation of smooth muscle cells within the *Tunica media* and an increase in the vessel lumen size^9^. Simultaneously, they undergo a structural renewal depending on trophoblast invasion^10^. In addition, the blood vessel system of the decidua expands by angiogenesis.

For the process of blood vessel remodeling and decidualization in addition to trophoblast invasion, the involvement of uterine natural killer cells (uNK) is of importance. These cells are especially located in the mesometrial lymphoid aggregate of pregnancy (MLAp), a tissue area, which is located between the muscle layers of the myometrium^11^. The MLAp was formerly known as the metrial gland^12^. Several studies report about uNK cell-depleted mice, which suffer from several pathological effects on placenta development, such as compromised spiral artery remodeling. However, no miscarriages or impairment of embryogenesis were reported in this context (reviewed in^13^).

An orchestrated action of transcription factors guarantees a balanced and progressive development and optimal function of many different organs, including the placenta. The basic helix-loop-helix (bHLH) transcription factor atonal homolog 8 (Atoh8), also known as murine Math6, has been implicated in a number of different biological functions in the mouse, zebrafish and human cultured cells. Thus, Atoh8 is associated with the specification and differentiation of cell lineages in neurogenesis and in the development of the kidney, pancreas and retina^14–17^.

Our previous studies showed that Atoh8 is also substantial for the regulation of myogenic progenitors during embryonic myogenesis^18^ as well as for the regeneration of human adult skeletal muscle fibers^19^. Analysis of *Math6* expression during murine embryonic development showed that this transcription factor may be involved in numerous processes during early and late development^20^. Investigations of the Atoh8 amino acid sequence in vertebrates reveal that the bHLH-domain is highly conserved across different species^21^ strongly emphasizing the importance of Atoh8.

Concerning the function of Math6 in mice, contradictory results have been published. Some years ago it was published that the lack of *Math6* gene expression caused by the deletion of genomic exon 1 and 2 in combination with intron 1 leads to an early embryonic lethal phenotype around the time of gastrulation^15^. Later it was shown that neither the single deletion of the first nor the second exon is responsible for this phenotype because newly generated constitutive homozygous *Math6* KO mice were born healthy and survive without any abnormalities^22^. These authors discussed that the initial method of *Math6* gene targeting in mice might have led to other unwanted genetic alterations than the *Math6* inactivation alone. These alterations might be responsible for the embryonic lethal phenotype of homozygous *Math6*^*EGFP-Cre*^ KO/reporter mice published by Lynn *et al*. in 2008.

Here we now describe the generation of a novel constitutive *Math6*^−/−^ mouse showing a completely new phenotype. By intercrossing heterozygous KO mice (*Math6*^+/−^) we are able to confirm that *Math6*^−/−^ mice develop normally and are born healthy and viable. However, *Math6*^−/−^ females are unable to fulfill a viable reproduction due to the occurrence of a variety of placenta abnormalities resulting in multiple miscarriages at late mid-gestation. Only a few placentas and embryos survive the hemorrhagic crisis and afterwards develop normally. However, fetuses could not be born most likely due to pathological alterations within the uterus caused by the aborted fetuses.

## Results

### Math6 deficient females are infertile

To analyze the systemic and organ-specific function of the transcription factor Math6 in mammals, we established a newly generated constitutive *Math6*^−/−^ mouse strain under the leadership of GenoWay incorporation. The murine *Math6* gene consists of three exons whereby exon 1 contains the translational start codon and codes for 257 of overall 322 amino acids. Exon 2 codes for 64 amino acids whereas exon 3 only contains the translational stop codon.

**Figure 1:**
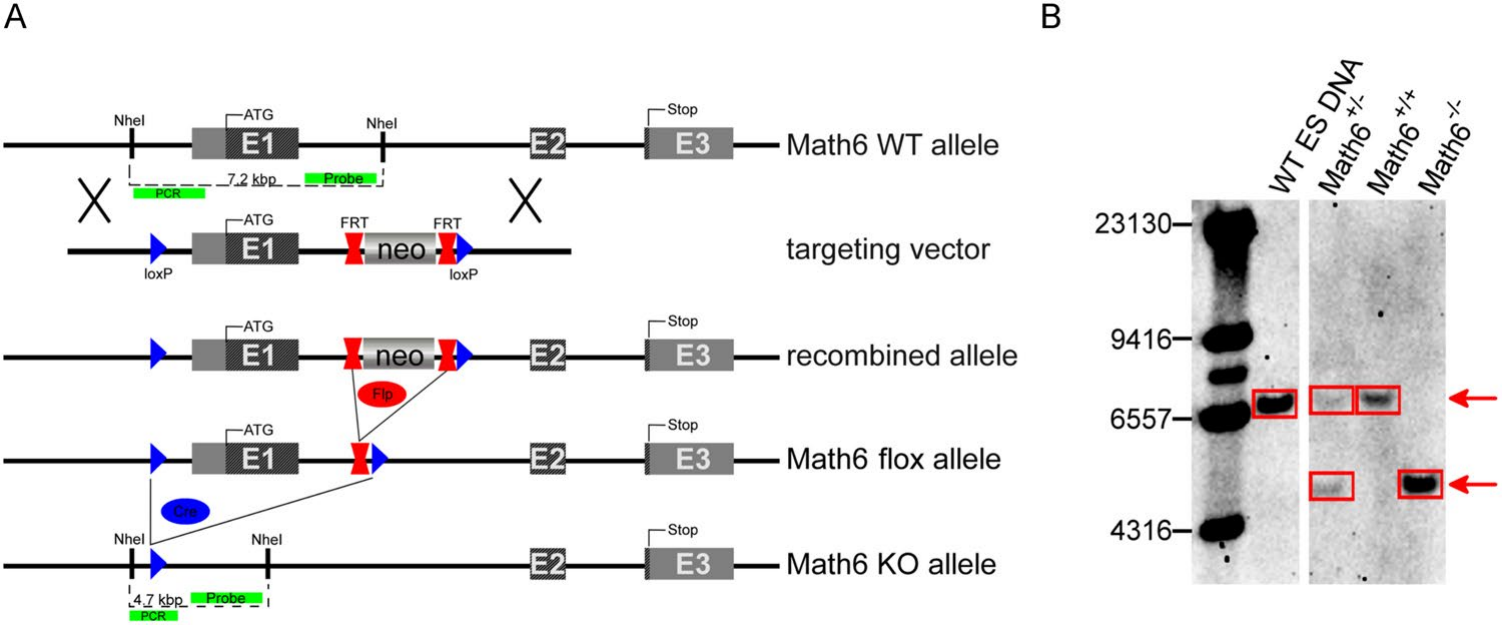
*Math6* gene targeting strategy. (**A**) Schematic drawing of the *Math6* WT allele, the gene targeting vector, the recombined *Math6* allele and the alleles resulting from the sequential deletion of the *Neo*-cassette (*Math6* flox allele) and *Math6* exon 1 (*Math6* KO allele) by Flp- and Cre-excision respectively. The long homology arm upstream of the 5′-flanking loxP-site is 5.9 kbp, whereas the short homology arm downstream of the 3′-flanking loxP site is 1.6 kbp in length. With respect to the *Math6* WT allele, the 5′-flanking loxP-site is located at position −1251 to −1217 with respect to the beginning of exon 1 (+1), whereas the 3′-loxP site is located at position +1389 to +1423. The locations of the DNA probe and PCR primers used for the detection of a successful gene targeting are indicated. (**B**) Southern Blot analysis of genomic DNA reveals the occurrence of all three *Math6* genotypes in the offspring of *Math6*^+/−^ intercrosses (WT allele: 7.2 kbp/KO allele: 4.7 kbp; picture shows cropped lanes of one blot cp. Fig. S8).

To achieve a functional KO, genomic *Math6* exon 1 was flanked by *loxP*-sites including the insertion of a *FRT*-flanked neomycin selection (*Neo*) cassette by classical gene targeting techniques in embryonic stem cells derived from C57Bl/6 J mice (Fig. 1). ES-clones with a homologous recombination event were selected and transferred to donor blastocysts of the albino C57BL/6J-Tyrc-2J/J mouse strain to generate chimeras.

Heterozygous *Math6*^*flox-FRT-Neo*/+^ mice derived from breedings of these chimeras with C57BL/6 wild type (WT) mice were crossed first with a *Flp-*deleter mouse to eliminate the *Neo*-cassette (*Math6*^*flox*/+^) and afterwards with a *Cre*-deleter mouse to remove exon 1 (Figs 1A and S1). Again, both deleter strains were of the C57BL/6 genetic background. Heterozygous *Math6*^+/−^ mice obtained were subsequently intercrossed. The genotype of their offspring distributed according to a Mendelian inheritance and *Math6*^−/−^ mice proved to be viable and healthy. The correct *Math6* gene deletion was confirmed by Southern Blot analysis (Fig. 1B) and the lack of any regular or truncated *Math6* mRNA was proven by reverse transcription quantitative PCR (RT-qPCR) (Fig. S2). Additionally, we analyzed the targeted *Math6* gene locus around exon 1 by partial genomic sequencing. For the phenotypic characterization and for the elimination of the *Flp*- and *Cre*-transgene the *Math6* KO allele was finally backcrossed into the C57Bl/6NJr genetic background for four generations and isogenic WT control mice were received in parallel.

When breeding *Math6*^−/−^ females with males of all three *Math6* genotypes we realized that homozygous KO females are unable to continue pregnancy to term independent from the paternal *Math6* genotype. Based on vaginal plugs and increasing abdominal girth of *Math6*^−/−^ females after mating, we concluded that infertility was not caused by a disrupted mating behavior or by early aborts around gastrulation like it was previously reported for constitutive *Math6*^*EGFP-Cre*^ KO/reporter mice^15^. In contrast, control breedings of *Math6*^+/−^ females with *Math6*^−/−^ males are productive and enable the preservation of the *Math6*^−/−^ mouse line (Fig. S1 and Table S1). These findings point to the conclusion that only the maternal *Math6* genotype determines the infertility observed in *Math6*^−/−^ females. This outcome is characterized by multiple miscarriages at late mid-gestation (E13.5) and only a few surviving fetuses at the time shortly before birth (E18.5). However, these fetuses could not be born although they are regularly developed. The type of infertility described for *Math6*^−/−^ females is very rare among genetically manipulated mice^23^ and hints to a so far undiscovered function of this transcription factor in mammals. According to our observations, we started to characterize the physiological function of Math6 in gestation in more detail.

### Elevated *Math6* gene expression in the placenta culminates at mid-gestation

Previous studies characterized the expression of *Math6* in several different organs. However, expression in the placenta is unknown so far. This led us to investigate the spatiotemporal expression of *Math6* in the placenta of WT mice by *in situ* hybridization (ISH) and RT-qPCR at four different time points during gestation. Placenta samples from *Math6*^−/−^ mice served as negative controls.

**Figure 2:**
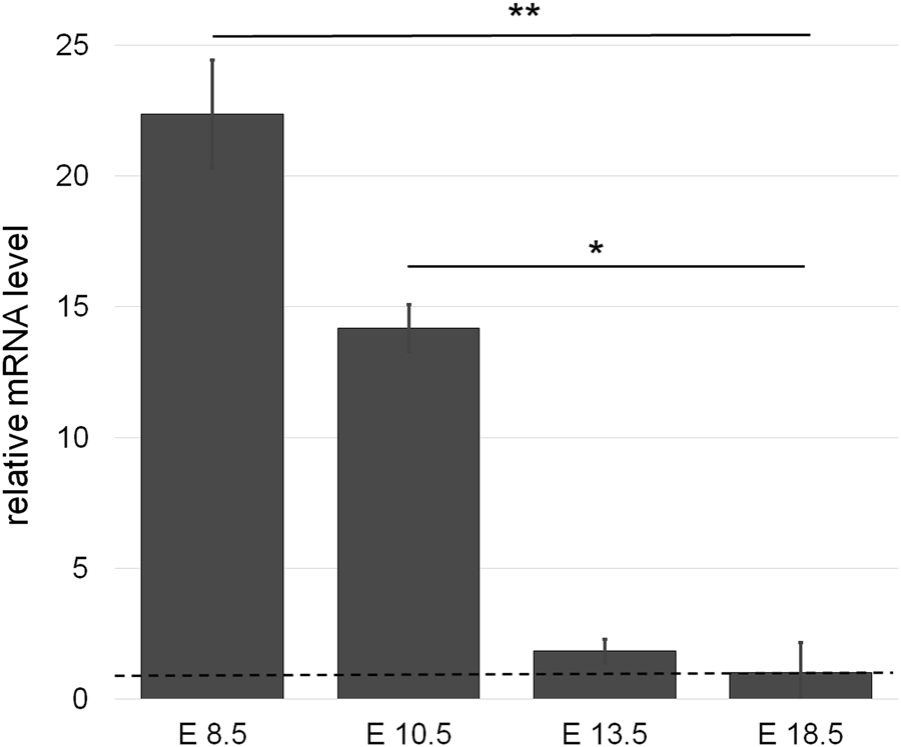
*Math6* gene expression in placenta tissue derived from pregnant WT mice. Relative mRNA levels are shown as fold changes to the reference day E18.5 (dotted line). Data are presented as mean ± SEM; n = 3 per time point.

Placental *Math6* was highly expressed at day E8.5 and E10.5 compared to the reference day E18.5, i.e. the time shortly before birth. The maximal mRNA expression was detected at day E8.5 where it is 22.4-fold higher than on the reference day (Fig. 2). On day E10.5 expression decreased although it is still 14.2 times higher than on the reference day. Afterwards the mRNA level drops rapidly to its lowest level at day E18.5. In short, *Math6* expression is high during early placenta development up to mid-gestation and afterwards decreases strongly to its lowest level shortly before birth (Fig. 2).

**Figure 3:**
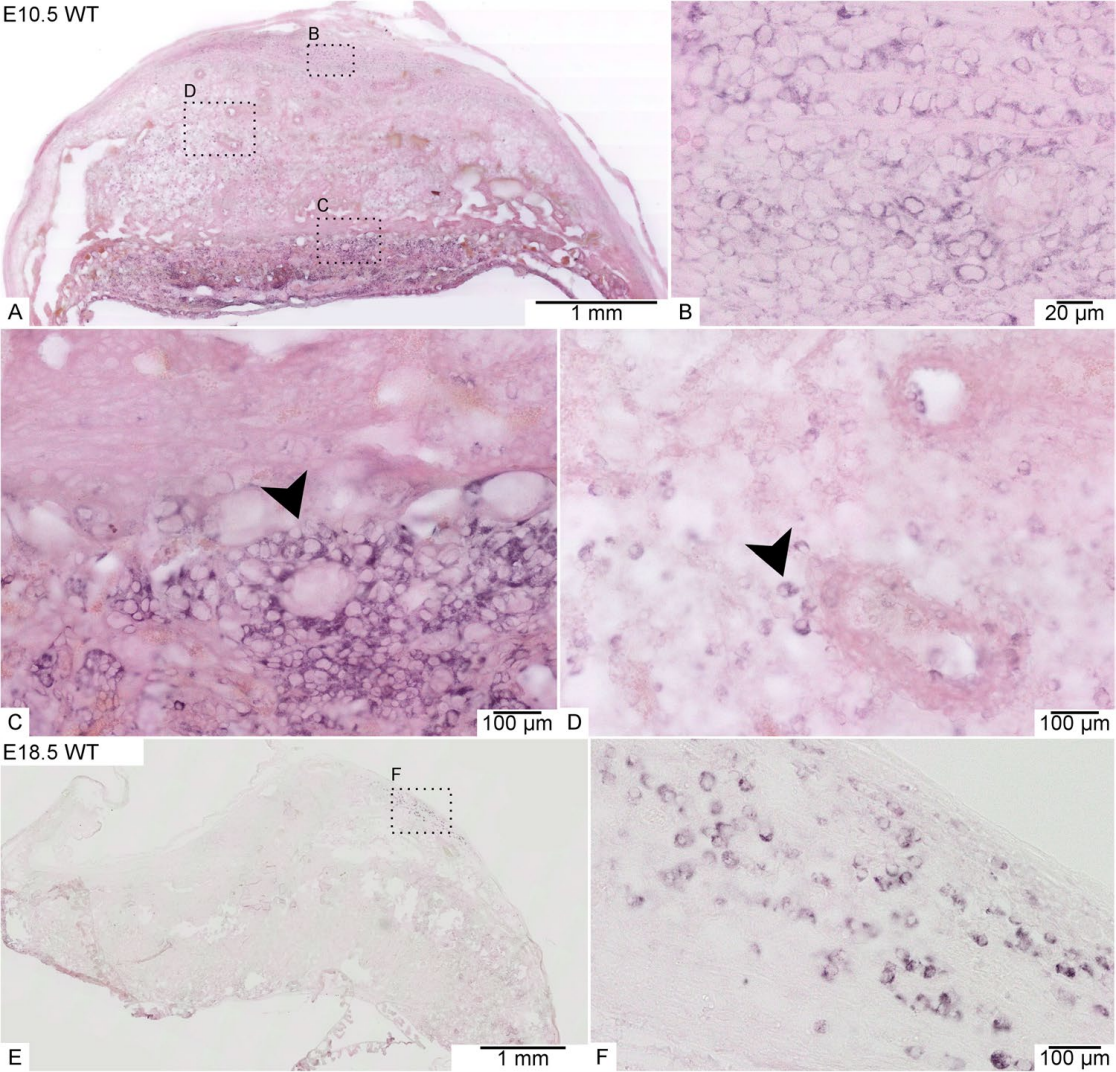
*Math6* gene expression analysis within the placenta of WT mice at day E10.5 (**A**–**D**) and day E18.5 (**E**,**F**) by ISH. At day E10.5 *Math6* mRNA occurred in the whole placenta, especially in spongiotrophoblasts of the junctional zone (C, arrowhead) and cells of the decidua (B + D), which to a part are located in direct contact to blood vessels (D arrowhead). At day E18.5 *Math6* gene expression was limited to only a few cells in the decidua (E + F).

To localize the spatiotemporal expression, we performed an ISH and investigated *Math6* mRNA transcription in different tissue layers and cell types of the placenta at analogous time points to the RT-qPCR analysis described before (Fig. 3). Unfortunately, it was not possible to detect the Math6 protein in our tissue samples by immunostaining and Western blot because of the lack of a specific antibody against murine Math6^24^. Our results reveal a strong *Math6* expression in all placental layers and a variety of cells at day E10.5 (Fig. 3A). On the one hand, we detected intensively stained cells in the maternal part of the placenta, i.e. in the MLAp as well as in the rest of the decidua, here often in association to the spiral arteries (Fig. 3B,D). These *Math6* expressing cells were identified as decidual and uNK cells. On the other hand, also trophoblasts in the junctional zone, especially spongiotrophoblasts, expressed *Math6* at high amounts (Fig. 3C), proving that both parts of the placenta, i.e. maternal and embryonic, harbor *Math6* expressing cells. At late stages of pregnancy, only a few cells still expressed *Math6* in the decidua. In accordance with our RT-qPCR results*, Math6* expression decreased with time of pregnancy and only persisted in a small cell population within the MLAp at day E18.5 (Fig. 3E,F).

### Maternal Math6 deficiency leads to an impaired placenta development

As the next step of phenotypic characterization, we performed a comparative investigation of placental development. Therefore, female mice of all three *Math6* genotypes were mated to either WT or *Math6*^−/−^ males and sacrificed at different stages of pregnancy. Gross anatomy of the gravid uterus was inspected and the placentas in common with the embryos/fetuses were resected and prepared for histological analysis. Morphometric evaluations on tissue sections, which were either stained by standard techniques or by immunostaining, were performed subsequently (Table S2).

Macroscopically visible alterations of the gravid uterus at day E8.5 and E10.5 between pregnant females of all *Math6* genotypes were not observed. However, in contrast to day E8.5 pathological alterations of the placentas derived from *Math6*^−/−^ females became obvious by microscopical investigation at day E10.5. Tissue sections of the implantation sites showed that especially the decidua was affected with respect to its vascularization, size and shape.

**Figure 4:**
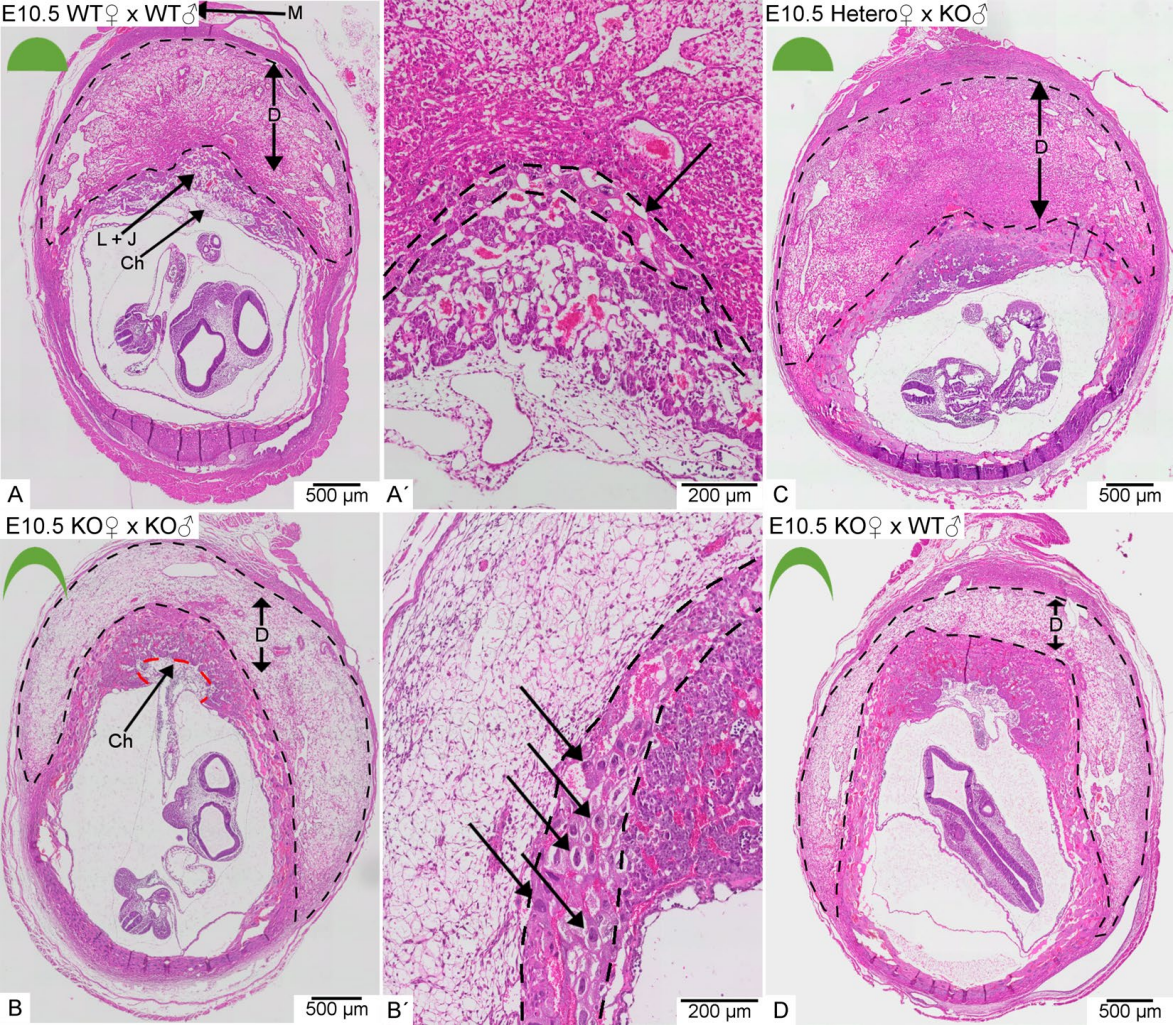
Morphology of placentas derived from WT in comparison to heterozygous (Hetero) or homozygous (KO) *Math6* deficient females at day E10.5. The decidua is located between the labyrinth + junctional zone (L + J) and the myometrium (M) and appeared semicircular in pregnant WT females (**A**). Complete (**B** and **D**) but not partial (**C**) lack of maternal Math6 led to an altered shape of the placenta with the junctional zone, labyrinth and chorionic trophoblast (Ch) convexly bulging into a crescent-shaped decidua (B/D). Additionally, the number of trophoblast giant cells in the junctional zone (black arrows) was increased in placentas lacking maternal Math6 (compare A′and B′). The placenta phenotype is independent of the paternal i.e. the resulting embryonic *Math6* genotype (compare B and D).

In WT intercrosses the placentas consisted of a large semicircular decidua and a fetal part built up by the junctional zone, the labyrinth and the chorionic trophoblast (Fig. 4A). In contrast, the placentas of *Math6*^−/−^ intercrosses are crescent-shaped with a fetal part convexly bulging into a flattened concave decidua at day E10.5 (Fig. 4B). The decidua appeared less eosinophilic and more loosely structured in HE-staining (Fig. 4B), although the number and morphology of decidual cells was not altered and they still expressed their marker desmin in a comparable way to WT placentas (Fig. S3). Consistent with an equal cell count we found no change in the proliferation rate of decidual cells by Ki-67 immunostaining (Fig. S4).

In contrast to the decidual cells, the number of trophoblast giant cells was increased in the junctional zone (Fig. 4B′). Interestingly, the same phenotype could also be observed in *Math6*^−/−^ females mated with WT males (Fig. 4D). This observation indicates that maternal *Math6* deficiency is sufficient to induce the pathological alterations described and that the embryonic *Math6* genotype is of minor or even of no importance for placenta development. Indeed, all placentas derived from *Math6*^+/−^ females mated with *Math6*^−/−^ males appeared like those of WT intercrosses (Fig. 4C).

**Figure 5:**
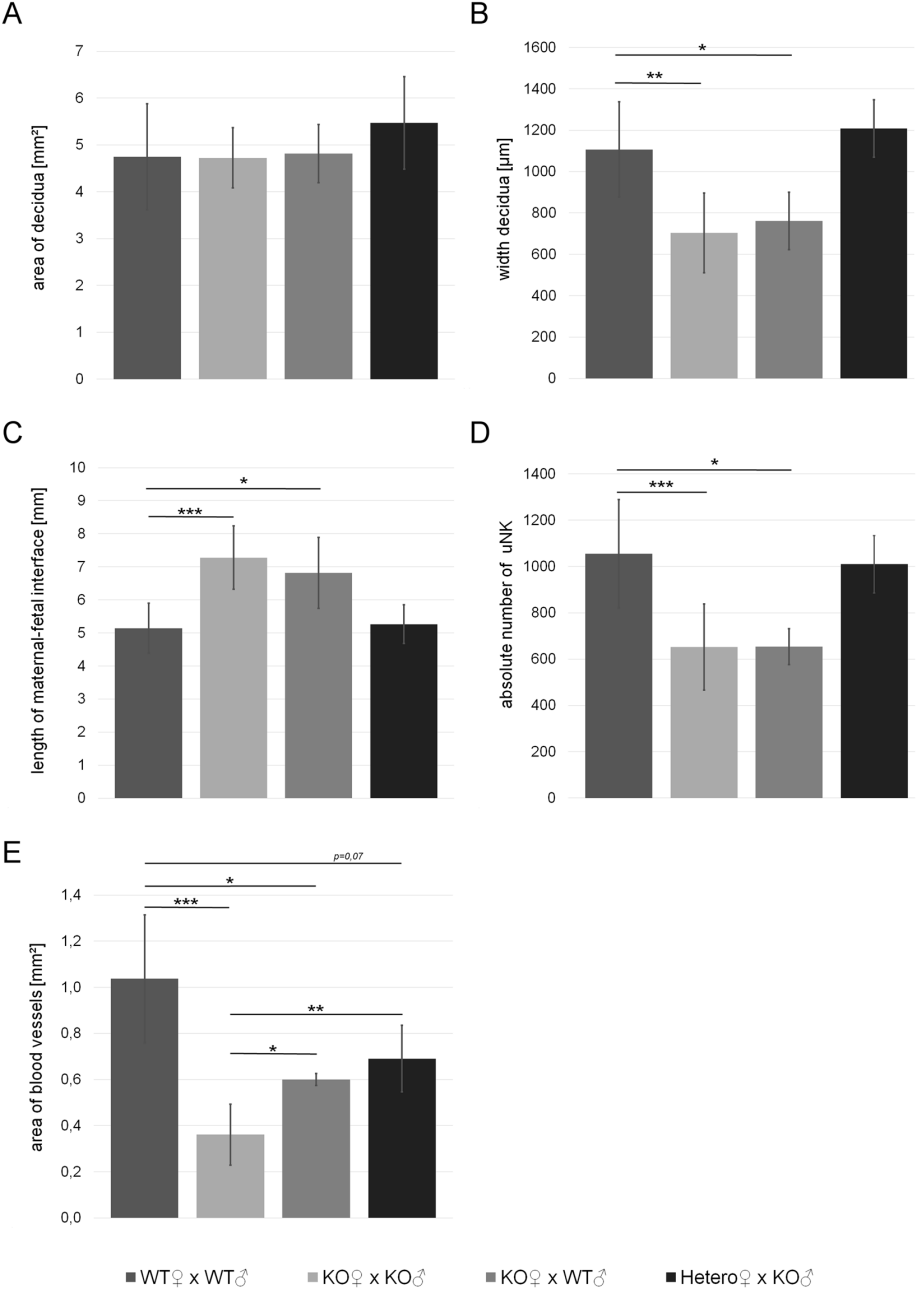
Morphology of the decidua in dependence of the parental *Math6* genotype. In contrast to the total area (**A**), the width of the decidua was significantly reduced in placentas derived from homozygous (KO) but not heterozygous (Hetero) females (**B**). This led to an enlargement of the length of the maternal-fetal interface in placentas derived from KO females (**C**). In addition, the absolute number of PAS-positive uterine natural killer cells (uNK, **D**) as well as the total area of the decidual blood vessel system (**E**) was reduced. Data are presented as mean ± SE; (WT × WT and KO × KO n = 7 and KO × WT n = 5; Hetero × KO n = 3). ***p ≤ 0.001 **p ≤ 0.01; *p ≤ 0.05.

Next we quantified parameters of decidualization in placentas dissected from the pregnant mice described above by performing morphometric analysis of HE-stained tissue sections on day E10.5. First, we measured the exact width of the decidua between the myometrium and the junctional zone and compared it to its total area. Although the width was significantly reduced in placentas derived from *Math6*^−/−^ in comparison to those from heterozygous or WT females, the total area remained approximately the same (Fig. 5A,B). These findings were accompanied by an altered shape of the decidua, i.e. the transformation from a semicircular to a crescent form which possesses an increased maternal-fetal interface (Fig. 5C). As mentioned above, the number of decidual cells was not altered in placentas derived from *Math6*^−/−^ mice, however, the number of uNK cells counted as total PAS-positive cells was significantly reduced (Figs 5D and S5). These cells are known to be necessary for remodeling spiral arteries and regulating decidualization. Consistently, we could prove that the maternal blood vessel system in the decidua was decreased, however, with no difference between arteries and veins. Therefore the vessel area was determined as a whole (Fig. 5E). In placentas derived from *Math6*^−/−^ intercrosses, the effect was pronounced with a highly significant reduction of about 65.2%. These data were confirmed by an immunostaining against alpha-smooth muscle actin (α-SMA) which is expressed in the *Tunica media* of the spiral arteries and which is considered as a marker for vascular integrity. The staining revealed a reduced number and size of arteries but disproved an altered vessel thickness (Fig. S6).

Interestingly and in contrast to all other parameters investigated, vascularization is partially recovered in placentas derived from *Math6*^−/−^ females mated to WT in comparison to *Math6*^−/−^ males. However, normal vascularization was not observed in breedings of *Math6*^+/−^ females mated to *Math6*^−/−^ males. These data hint to an impact of embryonic *Math6* expression on decidual vascularization and to the possibility that early placenta development occurs in a Math6 dose-dependent manner with respect to vascularization. Support for an existing gene dosage effect was obtained by RT-qPCR analysis of testis tissue, which possesses the highest *Math6* expression level in adult mice. The results show that both alleles are expressed, leading to a 2.6-fold decrease of *Math6* mRNA in *Math6*^+/−^ in comparison to WT males (Fig. S7).

At last, we intended to substantiate our histological analysis by investigating certain placental marker genes at day E10.5, i.e. the first time point of microscopically visible pathological alterations in all placentas of an individual *Math6*^−/−^ gravid uterus. We therefore performed RT-qPCR analysis comparing samples from *Math6*^−/−^ with those of WT intercrosses.

**Figure 6:**
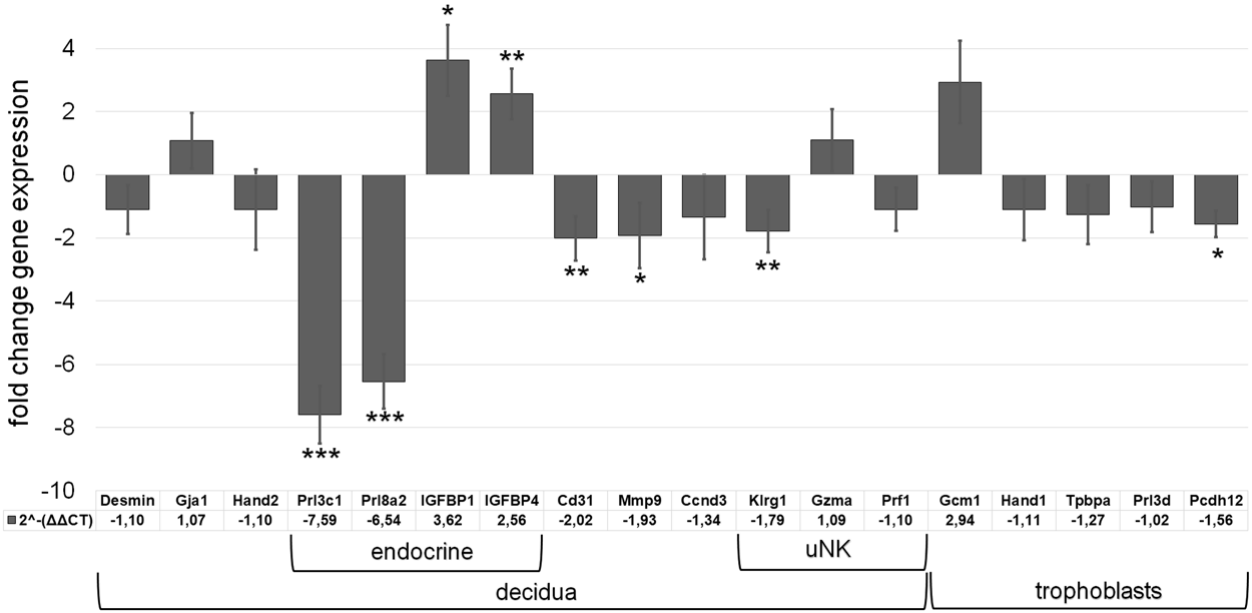
Influence of the *Math6* KO on the expression of marker genes in the placenta at day E10.5. While the mRNA levels of cellular decidua markers (Desmin, Gja1, Hand2) were not changed, the endocrine decidua markers (Prl3c1, Prl8a2, IGFBP1, IGFBP4) were altered significantly. Furthermore, mRNA levels of *CD31* (vascularization), *Mmp9* (extracellular matrix remodeling) and *Klrg1* (uNK cells) were significantly reduced whereas that of *Gcm1* (syncytiotropholasts) was increased. Data are presented as mean ± SEM; n = 6; *** ≤ 0,001 p**p ≤ 0,01; *p ≤ 0,05.

Consistent with the pathological signs observed in our histological examinations of placentas from *Math6*^−/−^ females, we found a significant two-fold decrease (Fig. 6) of platelet endothelial cell adhesion molecule 1 (PECAM1, CD31). In addition, also matrix metalloproteinase 9 (Mmp9) was 1.9-fold down-regulated (Fig. 6) indicating a certain combined defect of extracellular matrix remodeling and vascularization during decidualization in pregnant *Math6*^−/−^ females^25^.

As shown before by desmin and Ki67 immunostaining (Figs S3 and S4), defects of decidualization appeared not to be accompanied by an altered decidual cell count and proliferation. Indeed, decidual marker genes like desmin, Gja1 and Hand2 as well as the proliferation marker cyclin D3 (Ccnd3) were proven to be unchanged in addition. In contrast, paracrine acting endocrine factors of the prolactin family, which are known to be important for decidualization, like Prl8a2 (prolactin family 8 subfamily a member 2 or Dprp) and Prl3c1 (prolactin family 3 subfamily c member 1 or PLP-J)^26^ were strongly affected with a 6.5- and a 7.6-fold decrease in placentas from *Math6*^−/−^ mice (Fig. 6). Interestingly and in contrast to this, the insulin-growth factor binding proteins IGFBP1 and IGFBP4, which are paracrine regulators of trophoblast proliferation^27^, were found to be up-regulated by a 3.6- and 2.6-fold change, respectively. Concerning uNK cells we found contradictory results. Consistent with the reduced cell count detected by PAS staining (Fig. S5), expression of killer cell lectin-like receptor subfamily G member 1 (Klrg1) was reduced significantly whereas that of granzyme A (Gzma) and perforin 1 (Prf1) was unchanged.

At last, we determined, beside other trophoblast markers, the expression of the transcription factor glial cell missing 1 (Gcm1), a marker known to be exclusively expressed in syncytiotrophoblasts II and to be important for labyrinth development^28^. In contrast to the constant expression of the trophoblast markers Hand1, Tpbpa and Prl3d, Gcm1 is 3.0-fold up-regulated in placentas of *Math6*^−/−^ females.

### Maternal Math6 deficiency leads to miscarriages at late mid-gestation and infertility

The placental alterations in the uterus of *Math6*^−/−^ mice up to day E10.5 described so far led to terminal pathological consequences for most of the fetuses at later stages of pregnancy. Thus, during late mid-gestation at day E13.5, most of the pregnant *Math6*^−/−^ mice underwent a hemorrhagic crisis due to multiple miscarriages. To analyze these events in more detail, we started a second comparative study of pregnant females of all three *Math6* genotypes, which were mated again with either WT or *Math6*^−/−^ males. Females undergoing a life-threatening crisis were sacrificed at day E13.5, those which displayed a milder pathological outcome were investigated shortly before giving birth, i.e. on day E18.5.

**Figure 7:**
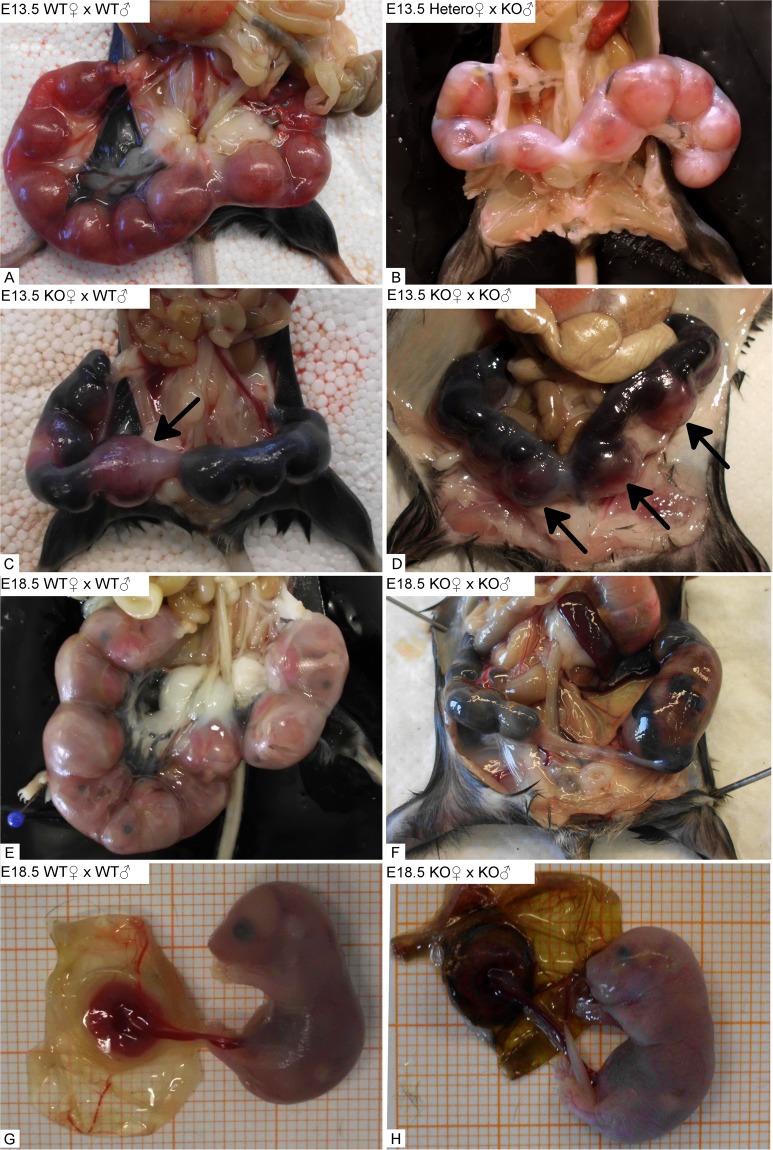
Gross anatomy of the gravid uterus at day E13.5 and day E18.5. Complete lack of maternal Math6 (KO) led to multiple uterine bleedings with fetal demise at most of the implantations sites in comparison and in contrast to heterozygous *Math6* KO (Hetero) and WT females (**A**–**D**). Only a limited number of placentas and fetuses were excluded from the general hemorrhagic uterine crisis (arrows) at day E13.5 and appeared to develop regularly up to day E18.5, i.e. the time shortly before birth (**E–H**). The remaining part of the uterus was filled with coagulated blood and necrotic tissue, which built up physical hindrances for the birth process.

On day E13.5 dissected females of WT intercrosses displayed a gravid uterus filled with a regular amount of well-developed implantation sites. The placentas within were correctly orientated towards the mesometrium, which appeared well vascularized by enlarged blood vessels (Fig. 7A). Consistent with our previous data, *Math6*^+/−^ females mated with *Math6*^−/−^ males displayed a pregnancy indistinguishable from that of WT intercrosses (Fig. 7B).

Indeed and in contrast to WT and heterozygous females, severe hemorrhagic events at most of the implantation sites occurred in pregnant *Math6*^−/−^ mice, which led to fetal demise. This hemorrhagic crisis took place independently of the paternal, i.e. the resulting embryonic *Math6* genotype (Fig. 7C,D). The diameter of the affected implantation sites appeared strongly reduced and black in color because the uterus itself, as well as the yolk sac, were filled with coagulated blood. No blood vessels were macroscopically visible in the mesometrial triangle at these sites. However, some fetuses were excluded from the hemorrhagic crisis and still seemed to be supplied by blood through a more or less functional placenta (Fig. 7C,D). These fetuses survive the general crisis at late mid-gestation like it was observed by dissection of pregnant *Math6*^−/−^ females at day E18.5 (Fig. 7E,F). Nevertheless even in these mice, blood clots in combination with necrotic tissue dominated at most of the implantation sites. These structures appeared as a physical hindrance for the birth process because born offspring from *Math6*^−/−^ females was never obtained, although the surviving fetuses were normally developed (Fig. 7G,H). To evaluate the degradation of the implantations, we analyzed the number of alive and dead fetuses by gross anatomical observation on day E13.5, E15.5 and E18.5 (Table S3). These data confirm that the number of alive implantations was reduced massively in *Math6*^−/−^ females.

To rule out a maternal lack or reduction of fibrinogen that would point to a blood clotting defect as a cause for spontaneous abortion^29,30^ we analyzed the concentration of fibrinogen-subunits in the blood plasma. There was no difference between the plasma fibrinogen levels or the amount of deposited fibrin within the placentas of WT and *Math6*^−/−^ females detectable (data not shown).

**Figure 8:**
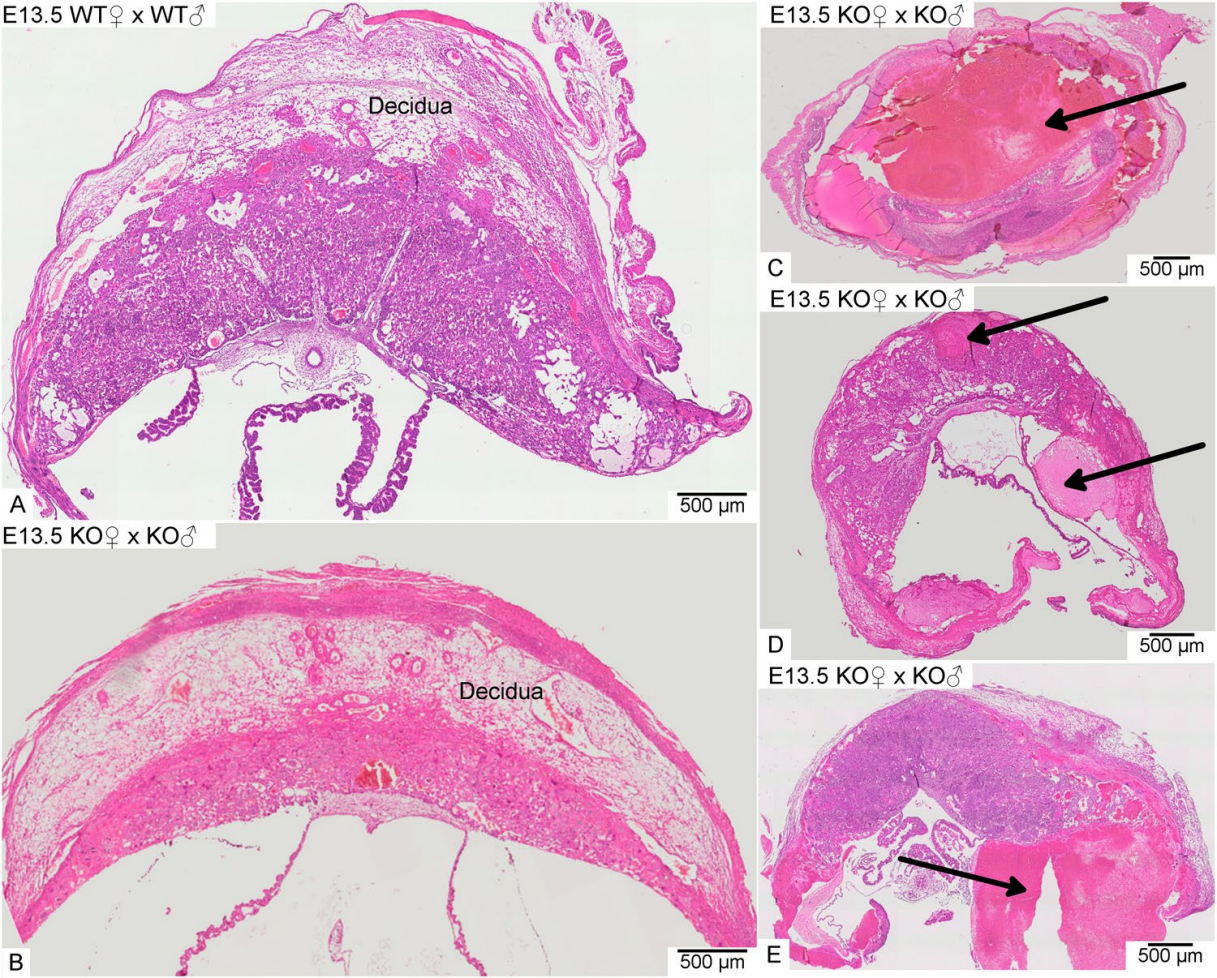
Exemplary HE-stained paraffin sections of placentas at day E13.5. In contrast to WT females (**A**), placentas dissected from an individual gravid uterus of a homozygous *Math6* KO female displayed a great variability of pathological alterations ranging from almost non-affected (**B**) to completely (**C**) or partially destroyed (**D** and **E**). Arrows point to blood clots.

Histological analysis at day E13.5 revealed a heterogeneous pathological pattern of placental and embryonic development in the gravid uterus of *Math6*^−/−^ females. Even in the same individual, the morphological variation was high. While in pregnant WT females all placentas were composed of distinguishable and well-developed tissue layers, in *Math6*^−/−^ females the placentas showed different degrees of size and tissue disintegration (Fig. 8). A very few implantation sites developed a placenta with a morphology comparable to the WT, i.e. all tissue layers were built up more or less regularly, although the decidua was clearly reduced in size (Fig. 8A,B). Such placentas have most likely supplied the fetuses surviving the general crisis at late mid-gestation and grew up until day E18.5. However, most of the implantation sites within an individual gravid uterus gave rise to placentas with a broad spectrum of pathological alterations. Thus, we found implantation sites with a single dominating blood clot and no detectable placental tissue anymore (Fig. 8C) in parallel to those, where clots occurred within or lateral to an irregular layered placenta with a flattened decidua (Fig. 8D,E). These placentas obviously did not give rise to any developed embryo or fetus.

## Discussion

The phenotype of *Math6*^−/−^ mice described in this manuscript unravels a so far undiscovered role of the transcription factor Math6 during placenta development in mammals. By generating a novel homozygous constitutive *Math6* KO mouse strain using *Cre-loxP*-mediated excision of exon 1 we were able to demonstrate that deficiency of Math6 leads to an irregular early development of all placentas within an individual gravid uterus up to day E10.5. Subsequently, severe complications of pregnancy occur in *Math6*^−/−^ females at late mid-gestation. Thus, multiple uterine bleedings took place around day E13.5 which led to a general hemorrhagic crisis and miscarriage of most of the fetuses. However, some placentas survived the time point of crisis, developed more or less regularly and supplied normally developed fetuses independently of the embryonic *Math6* genotype. This finding clearly demonstrates that Math6 is of no or minor function for mouse embryonic development itself, an observation, which is in clear contrast to the first publication of a constitutive *Math6* KO mouse described by Lynn *et al*. in 2008^15^. They report an embryonic lethal phenotype around gastrulation in heterozygous intercrosses of *Math6*^*EGFP-Cre*/+^ KO/reporter mice and never reached a homozygous status of their genetic manipulation with deletion of *Math6* exon 1, intron 1 and exon 2. In contrast, our generated *Math6*^−/−^ mice develop regularly and can be born by heterozygous but not by homozygous KO females. Thus, the maternal Math6 is of tremendous importance for a productive pregnancy.

In 2013, another group of authors including Lynn and colleagues withdrew their statement about embryonic lethality caused by Math6 deficiency^22^. They described different newly generated *Math6* KO mouse strains with a deletion of either exon 1, 2 or both in common with intron 1. However, for none of these strains they describe the phenotype presented by us in the present manuscript. Whether this is explained by different genetic backgrounds or whether they have not published the phenotype so far, remains unknown. Indeed, an influence of the murine genetic background on the phenotype of placenta development was previously published^31^. In the present manuscript the results were achieved within the inbred C57BL/6 genetic background, whereas Rawnsley *et al*. investigated mixed 129/SVxC57BL/6 mice.

Detailed histological as well as molecular biological analyses of placenta tissue derived from WT intercrosses revealed high placental expression of *Math6* in cells of the maternal as well as the fetal part of the early developing placenta at mid-gestation. Detection of *Math6* expression in decidual as well as in uNK cells in the maternal placentas thereby is in accordance with previous studies about the transcriptome of decidual cells showing high *Math6* expression in different cell subpopulations^32,33^. In contrast, *Math6* expression in the uterus and ovary of non-pregnant mice was very low, implicating its onset after fertilization or implantation (data not shown). However, during the early phase of placenta development, we found no difference in number and size of the implantation sites within the gravid uterus of WT and *Math6*^−/−^ females demonstrating that maternal Math6 is less important for implantation itself. Instead, it appears to regulate early placental development after implantation, particularly decidualization.

At day E10.5 the first microscopic alterations of the placental tissue composition and morphology became obvious between WT and *Math6*^−/−^ females. Although the total size of the decidua remained unaffected, its width was strongly reduced, the length of the maternal-fetal interface was extended and overall it appeared crescent-shaped. These changes might be caused by an irregular remodeling of the extracellular matrix in the decidua in combination with a reduced general vascularization. Consistently, genetic markers for both processes like *Mmp9* as well as *CD31* were down-regulated in parallel. It is well known that the depletion of Mmp9 expression in the mouse leads to an impaired pregnancy analogous to preeclampsia caused by defects in the maternal blood vessel system^34^ whereas CD31 is a prominent marker for the development and remodeling of blood vessels and is known to be highly expressed in the placenta on day E10.5^35^. For an optimal blood supply the decidual vessel system has to be expanded by angiogenesis and the spiral arteries have to be remodeled to low-resistance vessels^2^. Maternal uNK cells are known to regulate decidualization by their endocrine activity, especially by acting on spiral artery remodeling^13,36^. They were found to be decreased in the decidua of placentas from *Math6*^−/−^ females by histological analysis, an observation supported by a reduced expression of *Klrg1*, which is a receptor of mature uNK cells and a marker for active NK cell proliferation^37^. However, expression of *Gzma* and *Prf*, i.e. proteins of NK cell-mediated cytotoxicity, are not down-regulated. These contradictory results might reflect the high heterogeneity of the cell granule composition and the receptor repertoire between subpopulations of uNK cells^38^. Overall it has to be noted that a reduced amount of uNK cells cannot be the only cause for the irregular decidualization in pregnant *Math6*^−/−^ females since the morphological alterations found in the present study are not described for uNK cell depleted mouse models^36,39^. Thus, the lack of *Math6* expression within the decidual cells appears to have a higher or at least an additive impact on the phenotype.

By investigating this assumption in more detail we made some interesting findings. First, we found that the number of decidual in contrast to the uNK cells was not reduced. Thus, the intermediate filament desmin as a general decidual marker and the gap junction protein connexin 43, encoded by Gja1, which is important for trans-differentiation of stromal cells during decidualization^40^, were expressed regularly. The same result was achieved for the bHLH transcription factor Hand2, a marker for decidualization which is highly expressed in the uterine stroma^41^, as well as for the proliferation markers Ki67 and cyclin D3.

Secondly and in contrast to the number and proliferation status of decidual cells, we found that their endocrine function was strongly affected, as concluded from the tremendous alteration in the differential expression analysis of IGFBP1/2 as well as Prl3c1 and Prl8a2. In fact, IGFPB and prolactin-related proteins are inversely regulated, but both are important for the maternal-fetal communication by an endocrine process. Thus, controlled IGFBP secretion by decidual cells regulates trophoblast proliferation indirectly by buffering the bioavailability of insulin-like growth factor^27^. Furthermore, the importance of Prl3c1 and Prl8a2 for the overall endocrine function of the decidua was shown previously^42,43^.

In addition to the maternal placental cells, we detected *Math6* expression also in trophoblasts within the labyrinth and the junctional zone, here especially spongiotrophoblasts, at day E10.5. However, since the embryonic *Math6* genotype had no influence on the demise or survival of an embryo/fetus within *Math6*^−/−^ females, we conclude that it is of minor importance for the overall early placenta development, although it has to be noted, that embryonic *Math6* expression has a promoting effect on decidual vascularization. Indeed, it is known that other/further bHLH transcription factors like Mash2 and Hand1 regulate trophoblast proliferation and differentiation and therefore might compensate an endogenous lack of Math6^44,45^. In agreement with this assumption we found a regular expression of *Hand1* and of *Tpbpa, which is* expressed by spongiotrophoblast and glycogen trophoblasts in the junctional zone^46^.

In contrast, trophoblast giant cells of the junctional zone were found to be massively increased in number in placentas from *Math6*^−/−^ females, as shown by histological observation. However, this phenomenon occurred again independently of the embryonic *Math6* genotype and therefore appears to be caused predominantly by a maternal influence. Surprisingly, we found no up-regulation of *Prl3d*, a marker for trophoblast giant cells, to support their increased number. Therefore, we speculate that analogous to decidua cells, prolactin related proteins are also down-regulated in giant trophoblasts in response to the endogenous lack of Math6. However, the down-regulation might be masked by their increased cell count. Unfortunately, no further specific markers for giant trophoblasts are described so far to prove this hypothesis. Whether *Math6* expression in trophoblasts has a positive influence on the decidual vascularization, eventually by regulation of prolactin-related proteins, remains speculative.

At last, we tried to evaluate the status of the labyrinth by investigating *Gcm1*, a marker for syncytiotrophoblasts II which influences the labyrinth development^28^. Although the labyrinth appears to be built up regularly by histological investigation, the lack of Math6 expression leads to an increased expression of Gcm1. This phenomenon might reflect altered differentiation dynamics which have to be specified further.

Probably as a result of the altered decidua morphology and endocrinology in combination with the increased trophoblast giant cell number the junctional zone, the labyrinth and the chorion trophoblast bulge more convexly into the crescent-shaped decidua in placentas from *Math6*^−/−^ in contrast to those from WT females. Indeed, it is known that decidual cells normally inhibit trophoblast invasion by their endocrine activity^47,48^. Related to this, we hypothesize that *Math6* expression within decidual cells regulates a regular invasion, proliferation and differentiation of trophoblasts at the maternal-fetal interface during early placental development. The enhanced length of the interface in placentas from *Math6*^−/−^ females therefore appears to function as a compensatory mechanism for the altered paracrine cell communication or even reflects the need of a stronger mechanical stabilization. Alternatively, the unique placenta morphology might result from a deeper invasion of trophoblasts into the endometrium during implantation or from an irregular chorioallantoic fusion during early placentation.

Interestingly, we never found these morphological alterations in placentas from heterozygous females, demonstrating again that lack of *Math6* expression in the maternal and not the embryonic/fetal part is of major importance for them. The level of Math6 expression in the maternal part of the placentas from *Math6*^+/−^ mice thereby appears to be sufficient to guarantee an effectual ordered early placenta development and points to a dosage effect of Math6 on this process. Indeed, a gene dosage dependent reduction of *Math6* expression in *Math6*^+/−^ mice could be shown for the testis, which represents the tissue with the highest *Math6* expression detectable in adult mice.

To sum up, our data point to the conclusion that maternal Math6 is a main regulator of the endocrine activity during decidualization by altering the gene expression pattern of decidual cells on the one side and probably by decreasing the number of uNK cells or a subpopulation of them on the other side. Whether this reduction is a result of the altered endocrine activity of the decidual cells remains elusive. However, it appears obvious that the altered endocrinology causes an aberrant proliferation of trophoblast giant cells and an irregular maternal-fetal interface resulting in an overall disturbed placenta morphology. Since the major part of decidualization is known to take part between day E5.5 to E10.5, our spatiotemporal expression analysis of *Math6* fits well into the published literature.

At day E13.5 we found that most of the placentas within a gravid uterus showed bleedings with massive blood clot formation within the placenta tissue and yolk sack or around it. For some of the *Math6*^−/−^ females this crisis was life-threatening and we had to sacrifice them. However, some of them recovered from it within 24 h. The cause of these bleedings remains speculative but appears to be independent of reduced fibrinogen levels, which are known to be a considerable parameter of blood coagulation dynamics in association with pregnancy complications^29,30^. Instead, we suppose that they might be the result of the general irregular decidual vascularization and the disturbed formation of the maternal-fetal interface during early placental development. It is conceivable that the blood pressure within the mesometrial vessel system overwhelms the stability of the less developed vessel system within the individual placentas with time. This might have caused rupture of the vessels in most of them leading to an abrupt decrease in blood pressure and a general hemorrhagic crisis of the mouse. The resulting reduced nutrient and oxygen supply of the fetuses connected to the affected placentas might explain their demise. However, by random, some of the placentas and their fetuses were protected from the hemorrhagic crisis probably because of the reduced mesometrial blood pressure in consequence to the miscarriages of most of the others. This form of altruistic miscarriages could explain why the surviving placentas and fetuses might have recovered during the phase of reduced blood pressure. An alternative explanation could be a position-dependent blood flow in the murine uterus that is favoring some placentas due to their localization^49^. Thus placentas with the highest perfusion and/or with strongest impairment of their vessel system degrade whereas the others survive. However, this effect could not be affirmed in the *Math6*^−/−^ uterus so far (data not shown). In addition, we found that glycogen trophoblasts were not equally distributed in number within the placentas of an individual gravid uterus (data not shown). Therefore, the energy supply might vary between the different placentas during the hemorrhagic crisis resulting in an additional critical survival factor.

At day 18.5 pregnant *Math6*^−/−^ mice which recovered from the hemorrhagic crisis at late mid-gestation had to be finally sacrificed for humanitarian reasons because we realized that they were unable to give birth to the few surviving and normally developed fetuses. We speculate that this inability to give birth is the consequence of the pathological hemorrhagic events which took place during pregnancy. Thus, multiple blood clots in combination with tissue debris obstruct the uterus and thereby impair the birth process for the residual fetuses. However, we cannot exclude that further causes might participate in this unique form of infertility like for example an altered hormonal adaption^50^ in combination with an impaired myometrial function^51^.

Interestingly, to our knowledge, only one further mouse model, i.e. mice deficient for interleukin 11 receptor alpha (IL-11Rα), exists, which displays this unique form of infertility. These mice also suffer from uterine bleedings and miscarriages around day E10.5, which result from an affected decidualization with an increased number of trophoblast giant cells, a reduced number of differentiated uNK cells and an irregular remodeling of the extracellular matrix^23,52–54^. Although expression of IL-11Rα is not altered in *Math6*^−/−^ mice (data not shown), it remains to be elucidated whether the transcription factor Math6 is a down-stream effector of IL-11 Rα.

In summary, maternal Math6 is a critical factor for a sufficiently ordered early placenta development and influences vascularization as well as extracellular matrix remodeling within the decidua. In addition, Math6 determines trophoblast invasion, proliferation and differentiation at the maternal-fetal interface. Both functions are most likely be accomplished by a Math6 dependent endocrine activity of decidua as well as uNK cells, whereas Math6 expression in the trophoblast cells appears to be compensable by other/further factors. It will be of great interest whether Math6 deficiency in humans exists and whether it leads to recurrent miscarriages.

## Material and Methods

### Animals

Homozygous constitutive *Math6*^−/−^ KO mice were generated by a gene targeting strategy and procedure which was developed and performed by the GenoWay incorporation and which is described in the results section. All mice were fed *ad libitum* and kept under specific pathogen-free conditions in individually ventilated cages. All animal procedures performed in this work were in accordance with the German Animal Welfare Act and received prior acceptance by the Landesamt für Natur, Umwelt und Verbraucherschutz Nordrhein-Westfalen (No. 84-02.04.2014.A444).

### Southern Blot

The Southern Blot was performed according to a standard method^55^. Briefly, genomic DNA was isolated from mouse tails and hydrolysed with the *Nhe*I restriction enzyme. After agarose gel-electrophoresis the DNA was blotted onto a nylon membrane and hybridized with a 409 bp long, DIG-labeled probe. The probe was generated by PCR using DIG-labeled nucleotides. Labeling and the detection of the probe was performed using reagents from Roche Inc.

### Histological Analysis

Tissues samples were collected at different time points of pregnancy and fixed in 4% phosphate-buffered paraformaldehyde (PFA) for at least 24 h. Subsequently, the tissue samples were embedded in paraffin and sectioned with 7 µm thickness. The medio-sagittal sections of the radial symmetric shaped placentas showing the umbilical cord were stained with either hematoxylin and eosin (HE) or were treated according to the periodic-acid-schiff procedure (PAS staining). Stained sections were examined under the microscope and photographed using the virtual slide microscope VS120 (Olympus). Morphometrical analysis of/within the tissue sections was done using the Olympus OlyVia software. By assessing the histology of the placenta section, the blood vessels were identified in the decidua. Blood vessels appear as cell-free areas surrounded by an endothelium. The measurement tool of the OlyVia software was used to determine the blood vessel area by circumnavigate the area with a polygon. The surface area of the polygon was calculated by the software.

### RNA extraction and reverse transcription qPCR

Tissues were collected and immersed immediately with RNAlater (Qiagen) at 4 °C. Total RNA extraction was performed by the Trizol standard method (Invitrogen) and the subsequent cDNA synthesis was done using the QuantiTect Reverse Transcription Kit (Qiagen) and 1 µg of total RNA. Gene expression was detected using GoTaq qPCR Master Mix (Promega) for dye-based methods. The relative gene expression was analyzed by calculation of the 2^−ΔΔCT^ values and presented as relative fold changes^56,57^. The primer sequences are listed in the supplementary information Table S4.

### *In situ* hybridization

The protocol for ISH is a modified method described elsewhere in detail^58,59^. Briefly: Collected tissues were fixed in 4% phosphate-buffered PFA for 24 h following incubation in 30% sucrose in PBS overnight. Afterwards samples were embedded in OCT and frozen at −20 °C. Blocks were sectioned at a cryotome with 15 µm thickness. Received sections were fixed on superfrost glass slides for 1 h at 55 °C. To detect *Math6* mRNA expression by nonradioactive ISH, the digoxigenin-labeled specific anti-sense probe was produced by *in vitro* transcription with a DIG RNA labeling kit (Roche). The template is a 775 bp cDNA fragment of *Math6* cloned into the pDrive vector (Qiagen). Permeabilization of the tissue was performed with proteinase K (10 µg/ml) for 20 min at room temperature. Afterwards 1 µg/µl probe was hybridized for 15 h at 65 °C. The hybridized probe was colorimetrically detected by an anti-DIG antibody conjugated to alkaline phosphatase (Roche).

### Statistics

Statistical analysis was performed employing the unpaired two-tailed students-t-test. The number of samples (n) and the p-values of significant differences are given in each diagram respectively.

## Electronic supplementary material

Supplementary tables and figures
