# Supplementary material for: Murine transcription factor Math6 is a regulator of placenta development
Source: Sci Rep. 2018 Oct 9;8:14997. doi: 10.1038/s41598-018-33387-x (PMC6177472; doi:10.1038/s41598-018-33387-x)
Supplement: Supplementary file 1 — Supplementary tables and figures [file 41598_2018_33387_MOESM1_ESM.pdf]

Supplementary information

Title: Murine transcription factor Math6 is a regulator of placenta development

Marion Böing[1], Beate Brand-Saberi[1]*, Markus Napirei[1]

[1] Department of Anatomy and Molecular Embryology, Ruhr-University Bochum, Germany

*Corresponding author: Beate Brand-Saberi, Ruhr-University Bochum, Medical Faculty, Anatomy and Molecular Embryology, Building MA 5 Süd, R 158, Universitätsstr. 150, 44801 Bochum, Germany, Tel.: +49 (0)234 32 27780, Fax: +49 (0)234 32 14474, e-Mail: Beate.Brand-Saberi@rub.de

Table S1: Breeding statistics of *Math6* WT and KO mice.

***Math6* genotype**		**matings**	**litters**	**pups**	**pups / litter**
female	male				
+/+	+/+	9	25	168	6,7
+/-	-/-	10	25	140	5,6
-/-	+/+	6	0	0	/
-/-	-/-	6	0	0	/

Table S2: Placenta phenotype and reproductive rate in dependence of the parental *Math6* genotype (E10.5: +/+ x +/+ and -/- x -/- n=7 and -/- x -/- n=5; +/- x -/- n=3).

**parental *Math6* genotype**		**embryonic *Math6* genotype**	**morphology of the decidua at day E10.5**	**reproductive rate**
♀	♂	♀ **/** ♂		
+/+	+/+	+/+	normal morphology	viable offspring
+/-	-/-	+/-	normal morphology	viable offspring
		-/-		
-/-	+/+	+/-	altered shape, reduced uNK cells, impaired blood vessel remodeling	no offspring, uterine and vaginal bleeding
	-/-	-/-		

Table S3: Uterine implantation statistics in dependence of the maternal *Math6* genotype as evaluated by gross anatomical observation on day E10.5, E13.5 and E18.5.

**Maternal *Math6* genotype**	**total No. of implantations**	**total No. of alive implantations**	**mean No. of implantations**	**mean No. of alive implantations**
+/+	46	42	7,7	7,0
-/-	71	22	6,5	2,0

Table S4: Primer sequences

**Gene**	**Forward primer**	**Reverse primer**	**Ref.**
Desmin	CGAGGAGAGCAGGATCAACC	TCTCCATCCCGGGTCTCAAT	
Gja1	ATTTCGTTCCCGTGGAGGTG	TTTTGCCGCCTAGCTATCCC	
Hand2	CAAGATCAAGACACTGCGCC	TCTCCGTTCTGGTCGTCCTT	
Prl3c1	TGACTCAAGCACGCACCTG	TCCGGTCATATCGTGTGGCA	
Prl8a2	AGCCAGAAATCACTGCCACT	TGATCCATGCACCCATAAAA	57
IGFBP1	CATCCTGTGGAACGCCATCA	AGGGCTCCTTCCATTTCTTGAG	
IGFBP4	CTTCCACCCCAAACAGTGTCA	GTCTTCCGATCCACACACCA	
CD31	GCGGTGGTTGTCATTGGAGTG	GTTGGAGTTCAGAAGTGGAGCAG	57
Mmp9	CTCTGCTGCCCCTTACCAG	AGCGGTACAAGTATGCCTCTGC	
Ccnd3	CCTCCTACTTCCAGTGCGTG	GGCAGACGGTACCTAGAAGC	
Klrg1	AGGGAGTGAAGCTGTTTGGAG	CCTCCAGCCATCAATGTTCCT	
Gzma	CCTGAAGGAGGCTGTGAAAGAA	CGCCAGCACAGATGGTATTTG	
Prf1	TCTTGGTGGGACTTCAGCTTTC	TCTGCTTGCATTCTGACCGA	
Gcm1	TGCTCACCTATGGCTCTCCT	AAAATTCTGCCAAGCCCTTT	46
Hand1	GCAAGCGGAAAAGGGAGTTG	GGTGCGCCCTTTAATCCTCT	
Tpbpa	CCAGCACAGCTTTGGACATCA	AGCATCCAACTGCGCTTCA	46
Prl3d	TGGAGCCTACATTGTGGTGGA	TGGCAGTTGGTTTGGAGGA	46
Pcdh12	CCTAAGGGACTCTGCTCACG	CAGTACAGCCAGGCAGATCA	
Gapdh	CAGCCTCGTCCCGTAGACA	CGCTCCTGGAAGATGGTGAT	18
**Math6 Primer**			
Primer 1-2 (qPCR)	CGTCAATTTCACACGTAA	AAGAGACTCACAGTGGTGTCT	18
Primer 3-5	GTGCCGTGCTACTCCTATGG	CTGAGAGATGGTACACGGGC	
Primer 4-5	GAGCCAAGAAGCGGAAGGAG	CTGAGAGATGGTACACGGGC	
Probe	GTTTCTTTTTCCTAGCCCCAACCATTC	GGTATAATAAAGCATCCATACTGCCTTGTCC	
Genotyping WT	CATAAGGAGCCAGAGGTGCC	CTTCTACCTCCCACCCGAAC	
Genotyping KO	CATAAGGAGCCAGAGGTGCC	GCATACATTATACGAAGTTATATGCATCAC	

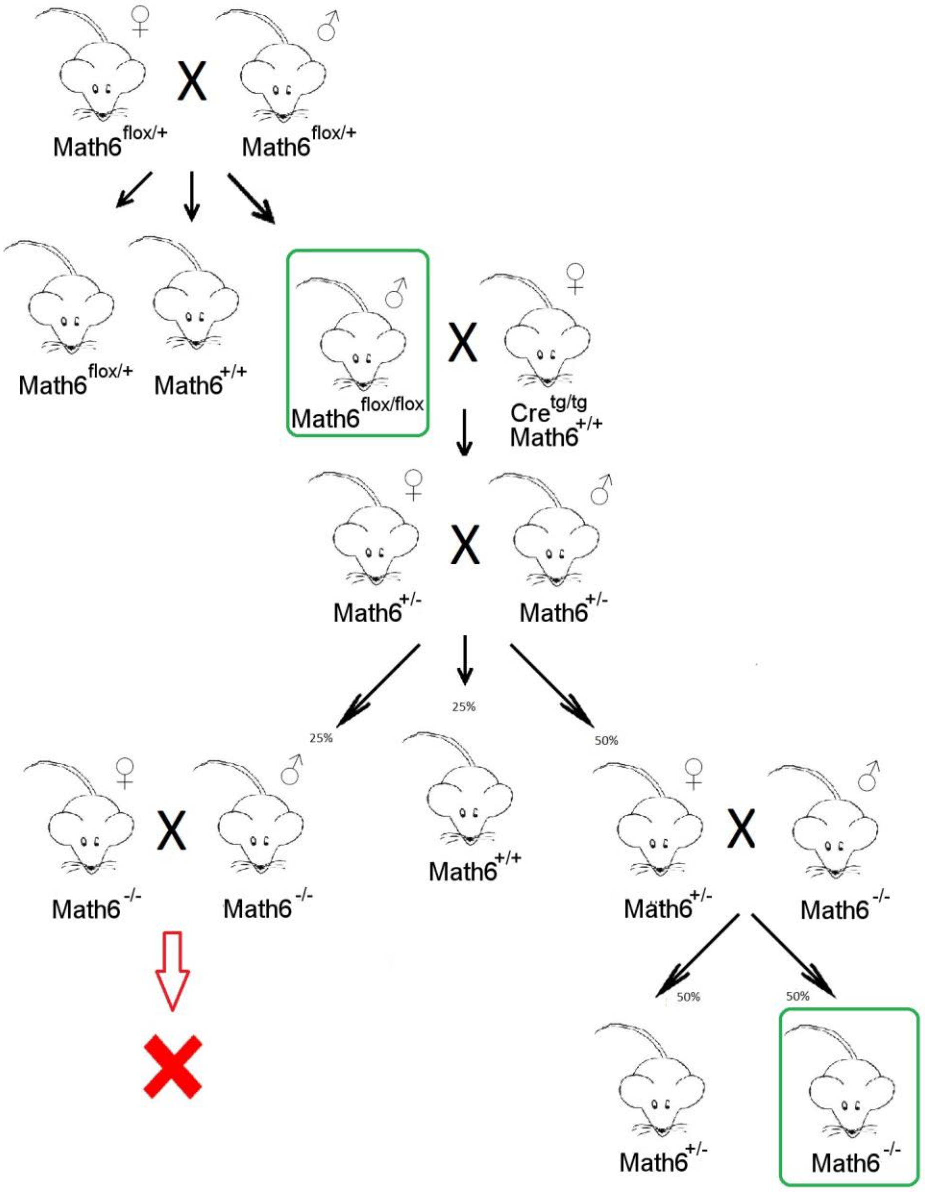

Figure S1: Breeding strategy of *Math6* $^{flox/+}$ mice (*Math6* exon 1 flanked by *loxP*-recombination sites) for receiving and maintaining homozygous *Math6* KO (*Math6*$^{-/-}$) mice. *Cre*$^{tg/tg}$ = "deleter" mouse homozygous for a ubiquitously expressed *Cre-recombinase* transgene.

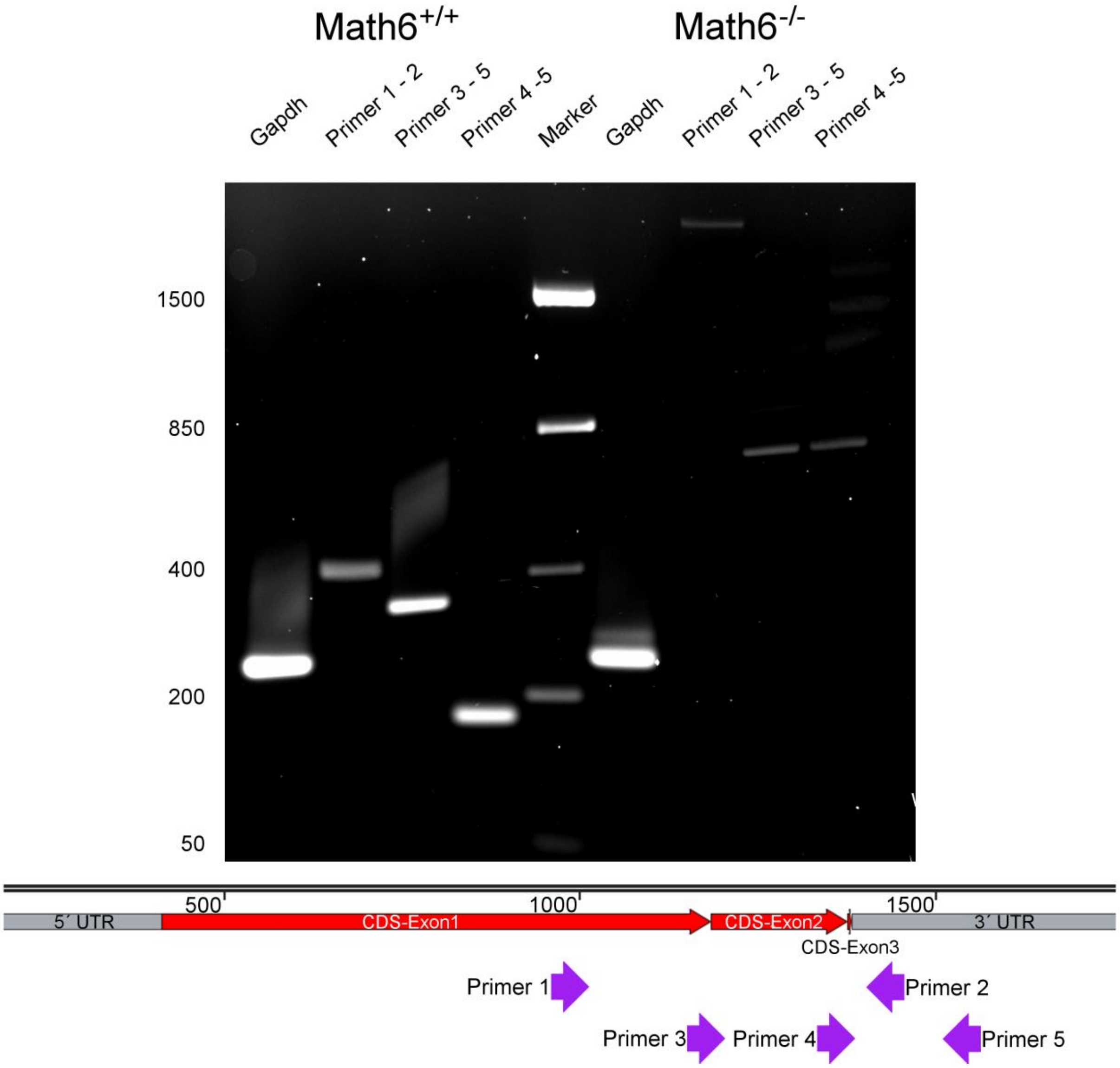

Figure S2: Representative gel-electrophoresis of *Math6* specific RT-PCR samples obtained from WT and KO mice. The localization of the primers used are indicated within the scheme of the *Math6* mRNA below the gel photo (length of the DNA amplificate with primers 1+2: 430 bp; primers 3+5: 346 bp; primers 4+5: 171 bp). In *Math6* KO mice no *Math6* related transcripts could be detected.

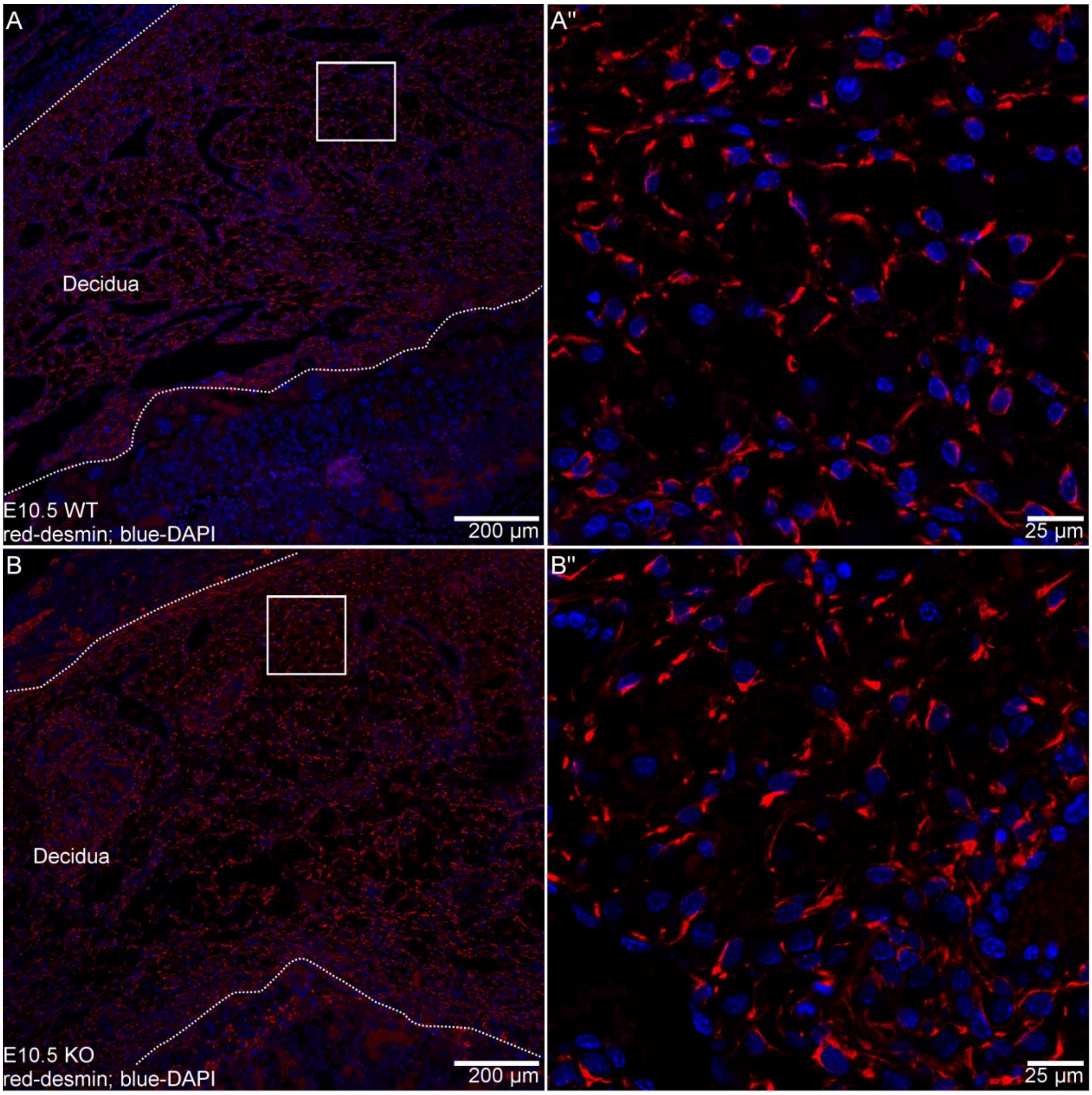

Figure S3: Qualitative analysis of decidual cells as detected by anti-desmin immuno- (red) and nuclear counterstaining by DAPI (blue). The amount of decidual cells is apparently not altered between placentas derived from WT in comparison to those from *Math6*$^{-/-}$ (KO) females.

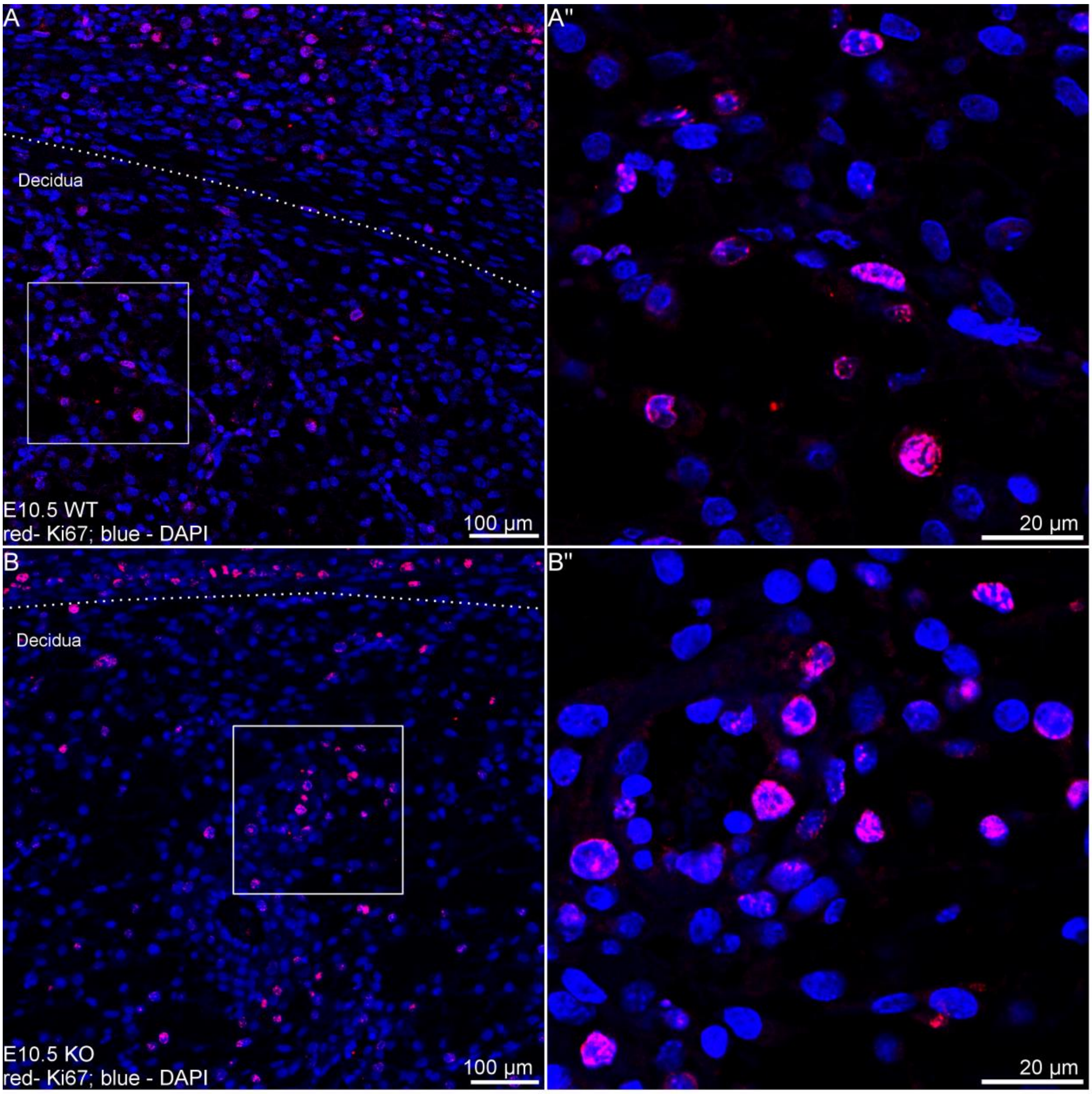

Figure S4: Qualitative analysis of proliferating decidual cells as detected by anti-Ki-67 immuno- (red) and nuclear counterstaining by DAPI (blue). The cell proliferation is apparently not altered between the maternal part of placentas derived from WT in comparison to those from *Math6-/-* (KO) females.

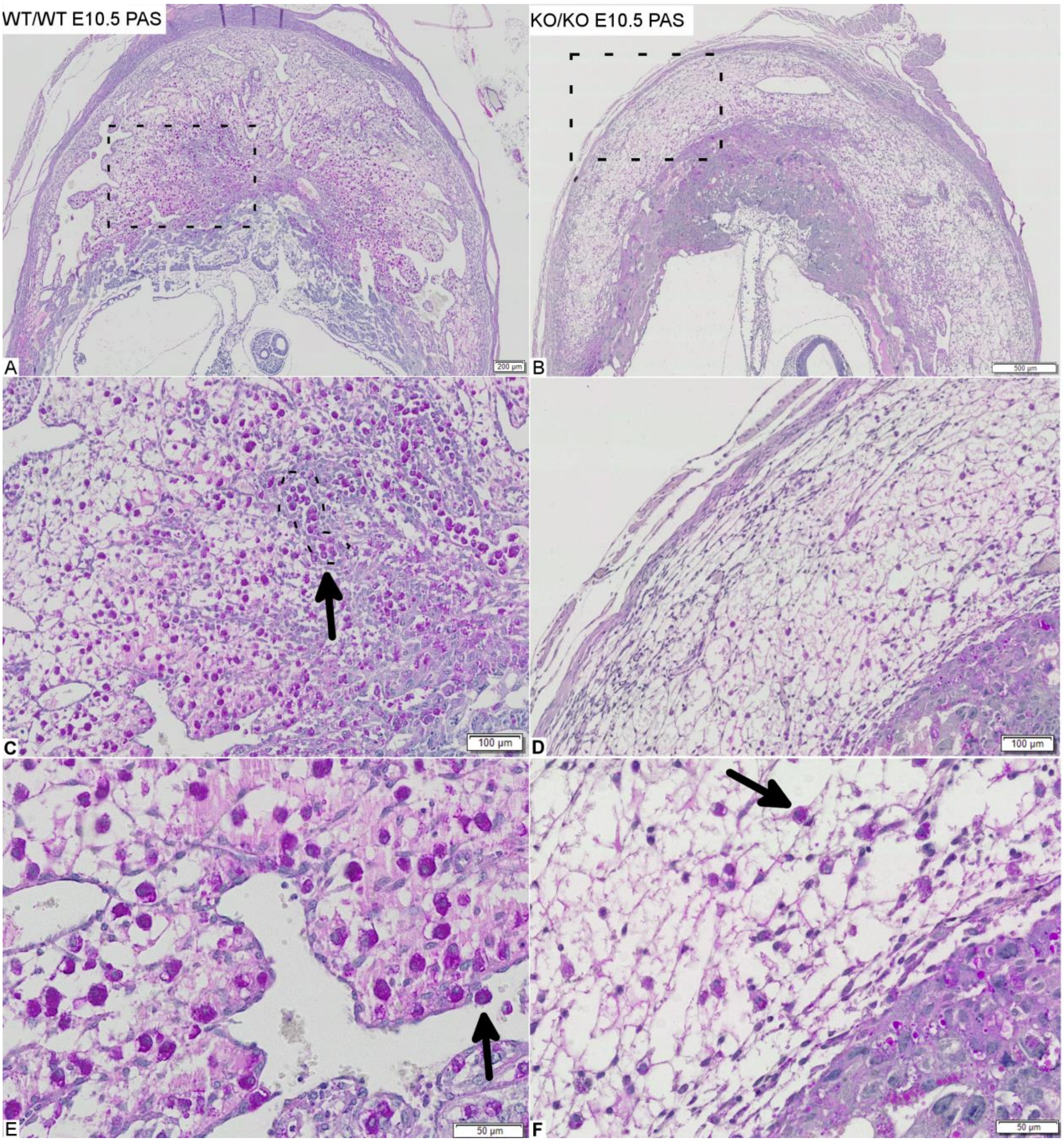

Figure S5: Qualitative analysis of uterine natural killer cells (uNK) in the placenta as detected by PAS-staining. The amount of uNK cells is apparently reduced in the decidua of placentas derived from *Math6-/-* (KO) in comparison to those from WT females.

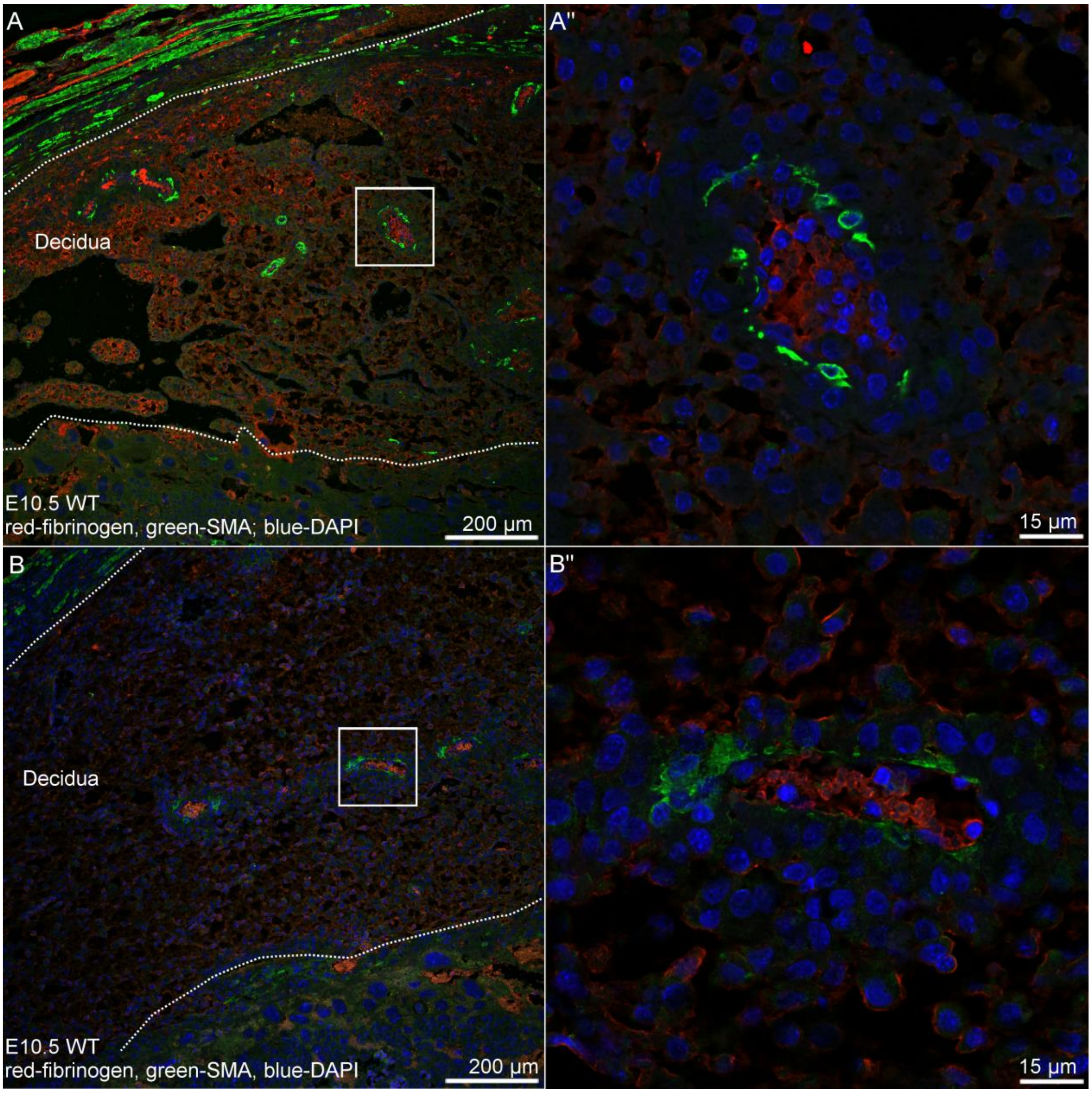

Figure S6: Qualitative analysis of spiral artery morphology detected by anti-alpha smooth-muscle-actin immuno- (green) and nuclear counterstaining by DAPI (blue). The anti-fibrinogen immunostaining (red) serves for better orientation within the sections. The amount of spiral arteries appeared reduced in the decidua of placentas derived from *Math6*$^{-/-}$(KO) compared to those from WT females. However, the *Tunica media* of these arteries was apparently not altered.

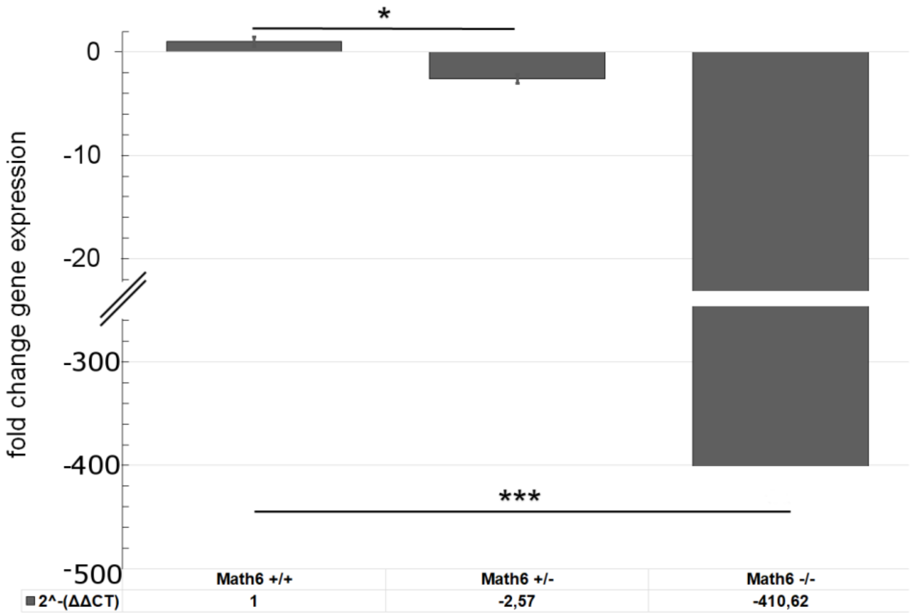

Figure S7: Detection of a *Math6* gene dosage effect with respect to the expression level in the testis of mice of all three genotypes. Heterozygous *Math6* gene expression leads to a 2.57-fold reduction of the mRNA level, whereas in homozygous KO mice the expression level was completely abrogated. Data are presented as mean ±SEM; n=3; ***≤ 0,001 p; * p≤ 0,05

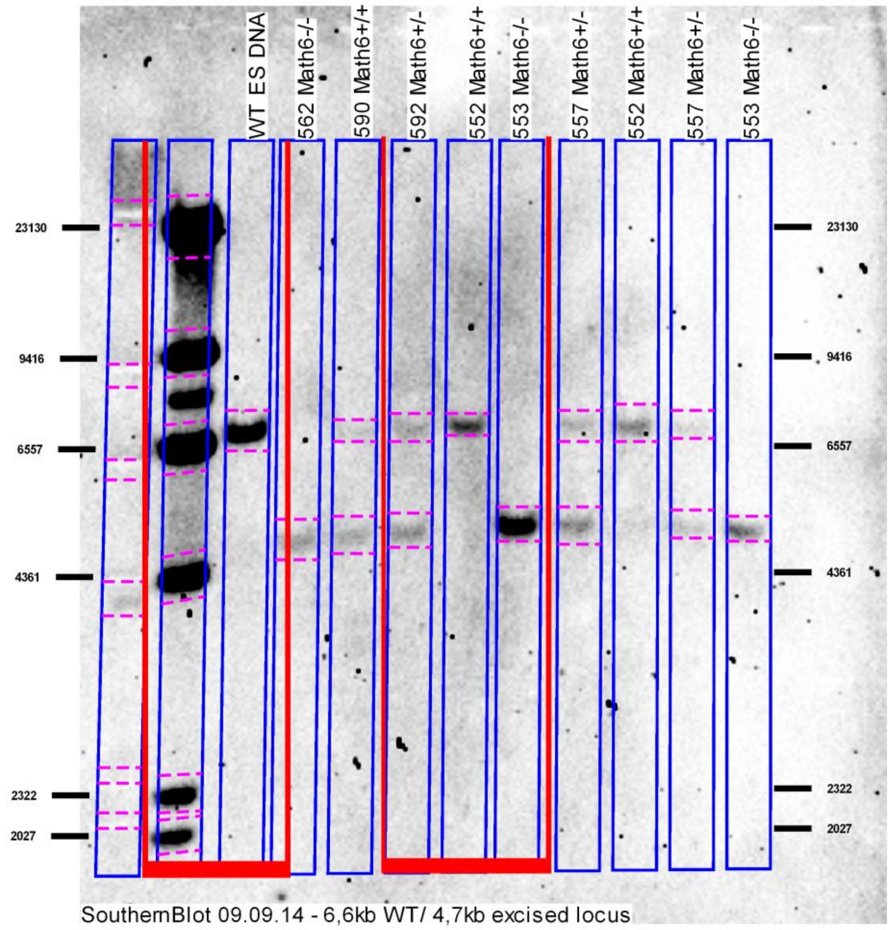

Figure S8: Full picture of the Southern Blot, which is partially presented in Fig.1B.
